# Supplementary material for: Screening microbially produced Δ9-tetrahydrocannabinol using a yeast biosensor workflow
Source: Nat Commun. 2022 Sep 20;13:5509. doi: 10.1038/s41467-022-33207-x (PMC9489785; doi:10.1038/s41467-022-33207-x)

Supplementary Information for

Screening microbially produced Δ9-tetrahydrocannabinol using a yeast biosensor workflow

William M. Shaw[1,2,3,4], Yunfeng Zhang[5], Xinyu Lu[3,4], Ahmad S. Khalil[1,2,6], Graham Ladds[7], Xiaozhou Luo[5] and Tom Ellis[3,4*]

[1]Biological Design Center, Boston University, Boston, MA 02215, USA

[2]Department of Biomedical Engineering, Boston University, Boston, MA 02215, USA

[3]Department of Bioengineering Imperial College London, London, SW7 2AZ, UK

[4]Imperial College Centre for Synthetic Biology, Imperial College London, London, SW7 2AZ, UK

[5]Center for Synthetic Biochemistry, Shenzhen Institute of Synthetic Biology, Shenzhen Institute of Advanced Technology, Chinese Academy of Sciences, Shenzhen, 518055, China

[6]Wyss Institute for Biologically Inspired Engineering, Harvard University, Boston, MA 02115, USA

[7]Department of Pharmacology, University of Cambridge, Cambridge CB2 1PD, UK

*Corresponding author: Tom Ellis t.ellis@imperial.ac.uk

This Supplementary Information includes:

Supplementary Fig. 1. Screening the cannabinoid GPCR/Gα variant strains against endogenous agonists.

Supplementary Fig. 2. Receptor independent effects of 2-AG.

Supplementary Fig. 3. CB2 biosensor dose-response curves with cannabinoids and their precursors.

Supplementary Fig. 4. Receptor-independent effects of cannabinoids and precursors.

Supplementary Fig. 5. CB2 biosensor plate reader assay optimisation.

Supplementary Fig. 6. Properties of the oxidised and variant cannabinoids, cannabinol (CBN), cannabigerovarin (CBGV), Δ9-tetrahydrocannabivarin (THCV), and cannabivarin (CBV).

Supplementary Fig. 7. Decarboxylation of THCA producing yeast cell extract.

Supplementary Table 1. THCAS mutant strains in the yS231 library.

Supplementary Table 2. Ligands used in this study.

Supplementary Table 3. DNA sequences used in this study.

Supplementary Note 1. Gating strategy for flow cytometry.

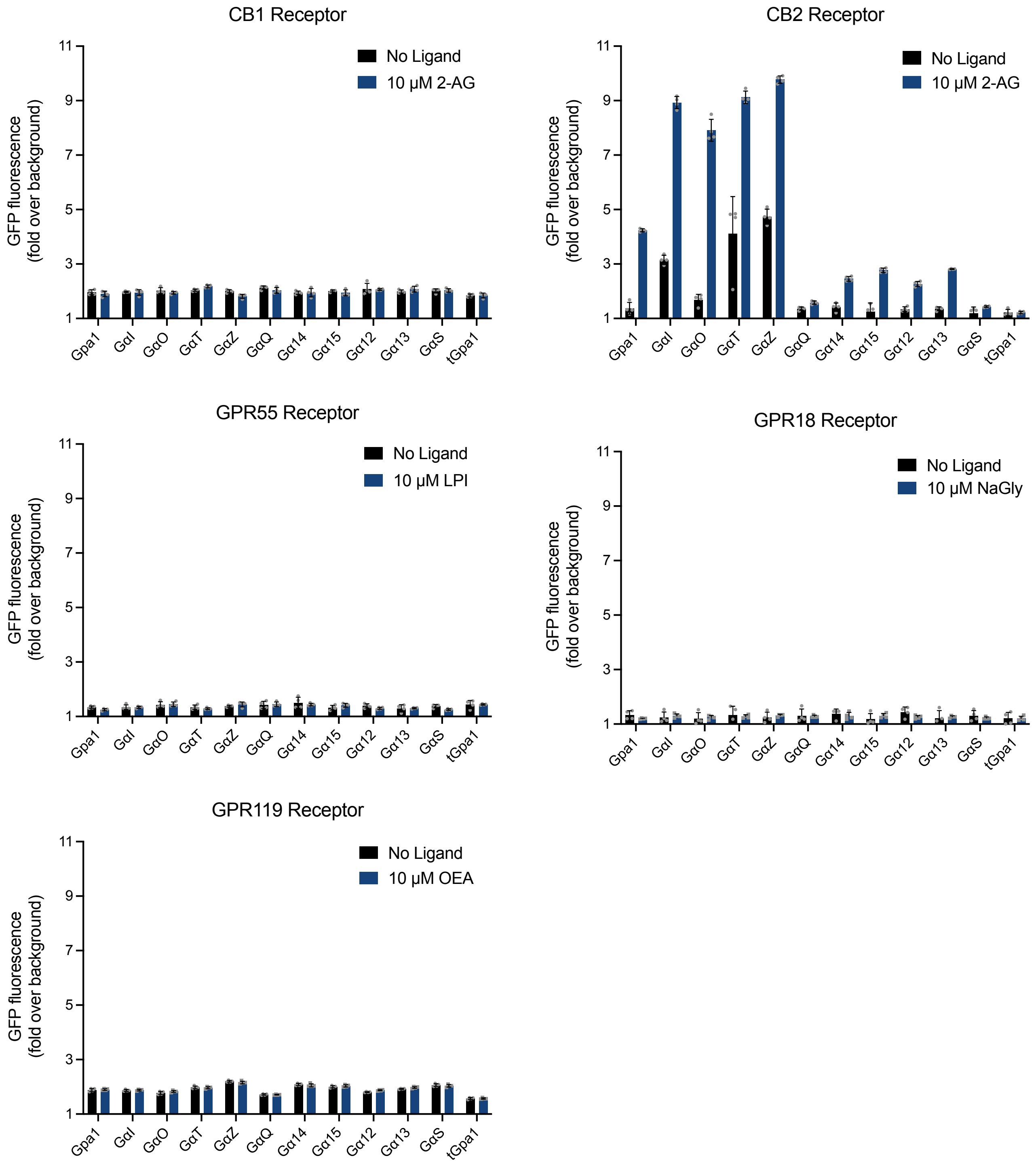

Supplementary Fig. 1. Screening the cannabinoid GPCR/Gα variant strains against endogenous agonists. ON/OFF response of the 60 GPCR/Gα strains to 10 µM of the endogenous agonists 2-AG (CB1R and CB2R), NaGly (GPR18), LPI (GPR55), and OEA (GPR119). Experimental measurements are GFP levels per cell as determined by a plate reader and shown as the mean ± SD from four biological replicates. Note that the small but observable differences in GFP expression after addition of ligand in CB1R and GPR18 yeast disappear upon normalising for receptor-independent changes in **Fig 1c**, which are also seen in the tGpa1 control.

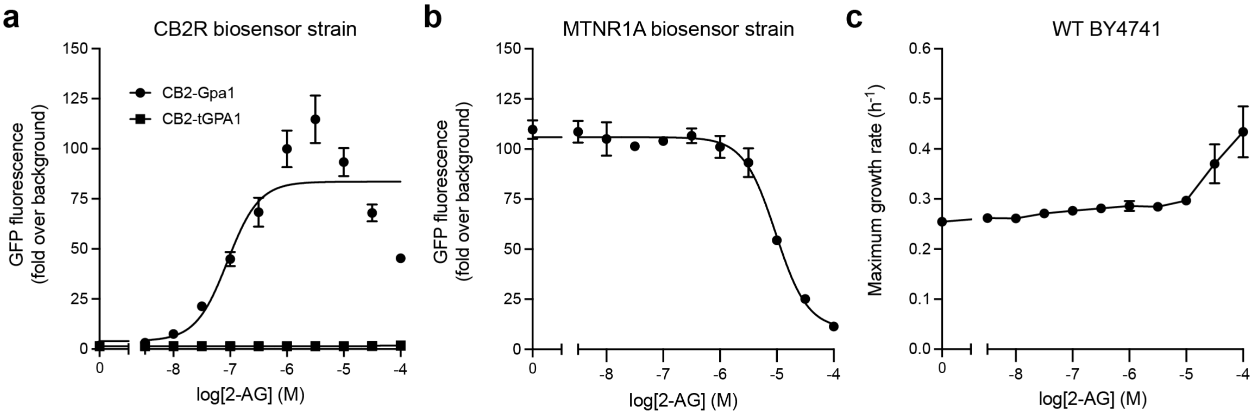

Supplementary Fig. 2. Receptor independent effects of 2-AG. **a**, 2-AG dose-response curves with the CB2R/Gpa1 biosensor (circles) and the CB2R/tGpa1 control (squares). **b**, 2-AG dose-response curves with a MTNR1A biosensor strain from Shaw et al. at the half maximal-effective concentration (EC_{50}) of melatonin (0.3 µM). Receptor-independent effect on reporter output seen above 1 µM 2-AG, possibly explaining the non-sigmoidal dose-response curve in **a**. Experimental measurements are GFP levels per cell determined by flow cytometry and shown as the mean ± SD from four biological replicates. Curves were fitted using GraphPad Prism variable slope (four parameter) nonlinear regression fit. **c**, Maximum growth rate of wildtype BY4741 yeast over a range of 2-AG concentrations. Interestingly, an increase in growth rate is seen above 1 µM 2-AG. Measurements are maximum growth rate calculated from growth at exponential phase in YPD and shown as mean ± SD from four biological replicates for a and b, and eight biological replicates for c.

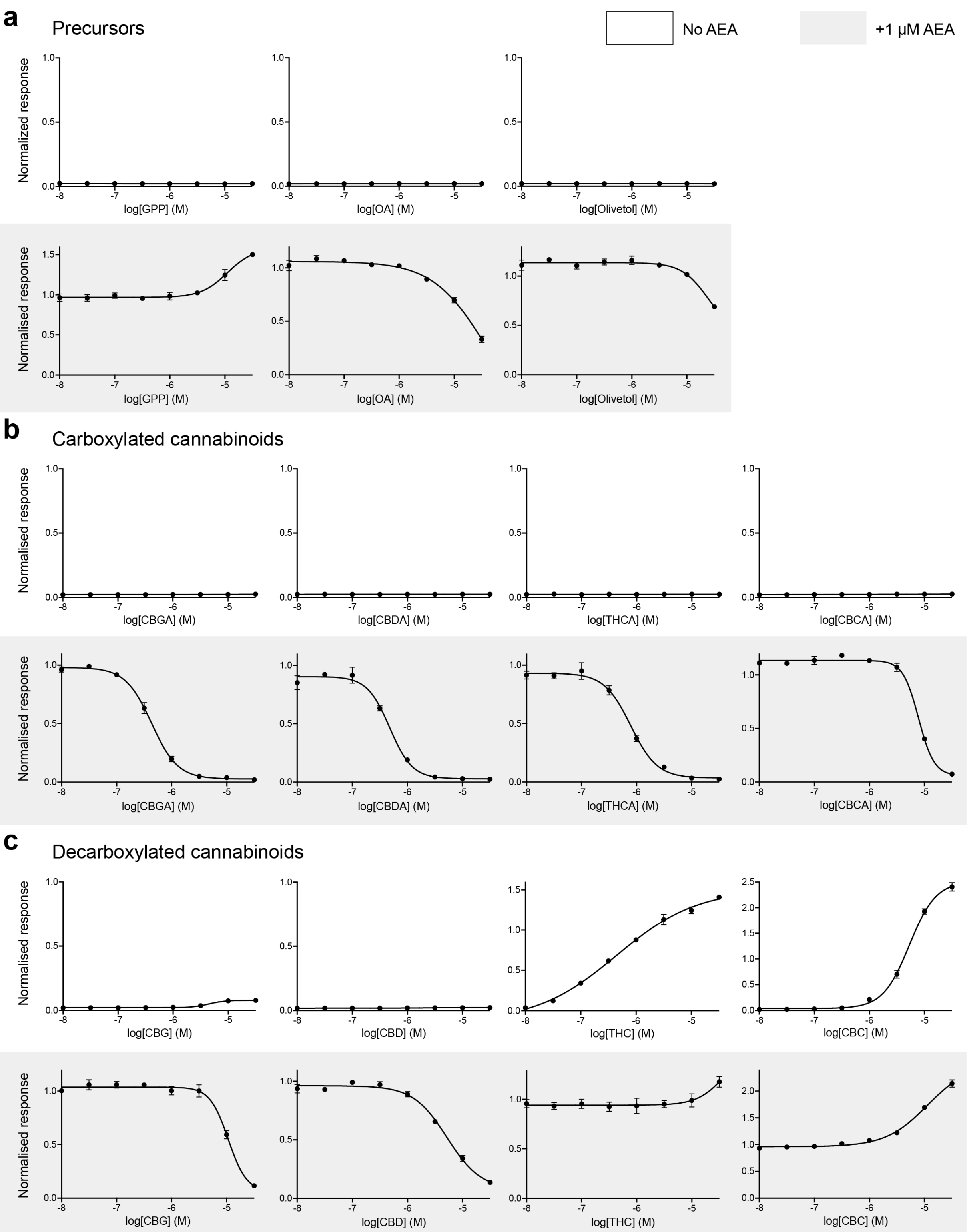

Supplementary Fig. 3. CB2 biosensor dose-response curves with cannabinoids and their precursors. a, Dose-response curves of the cannabinoid biosynthesis precursors in the presence (grey shade) and absence (no shade) of 1 µM AEA**. b**, Dose-response curves of the cannabinoid acids in the presence (grey shade) and absence (no shade) of 1 µM AEA**. c**, Dose-response curves of the decarboxylated cannabinoids in the presence (grey shade) and absence (no shade) of 1 µM AEA**.** Experimental measurements are GFP levels per cell determined by flow cytometry and shown as the mean ± SD from three biological replicates. All data are normalised to the no ligand (0) and 1 µM AEA (1) CB2 biosensor response. Curves were fitted using GraphPad Prism variable slope (four parameter) nonlinear regression fit.

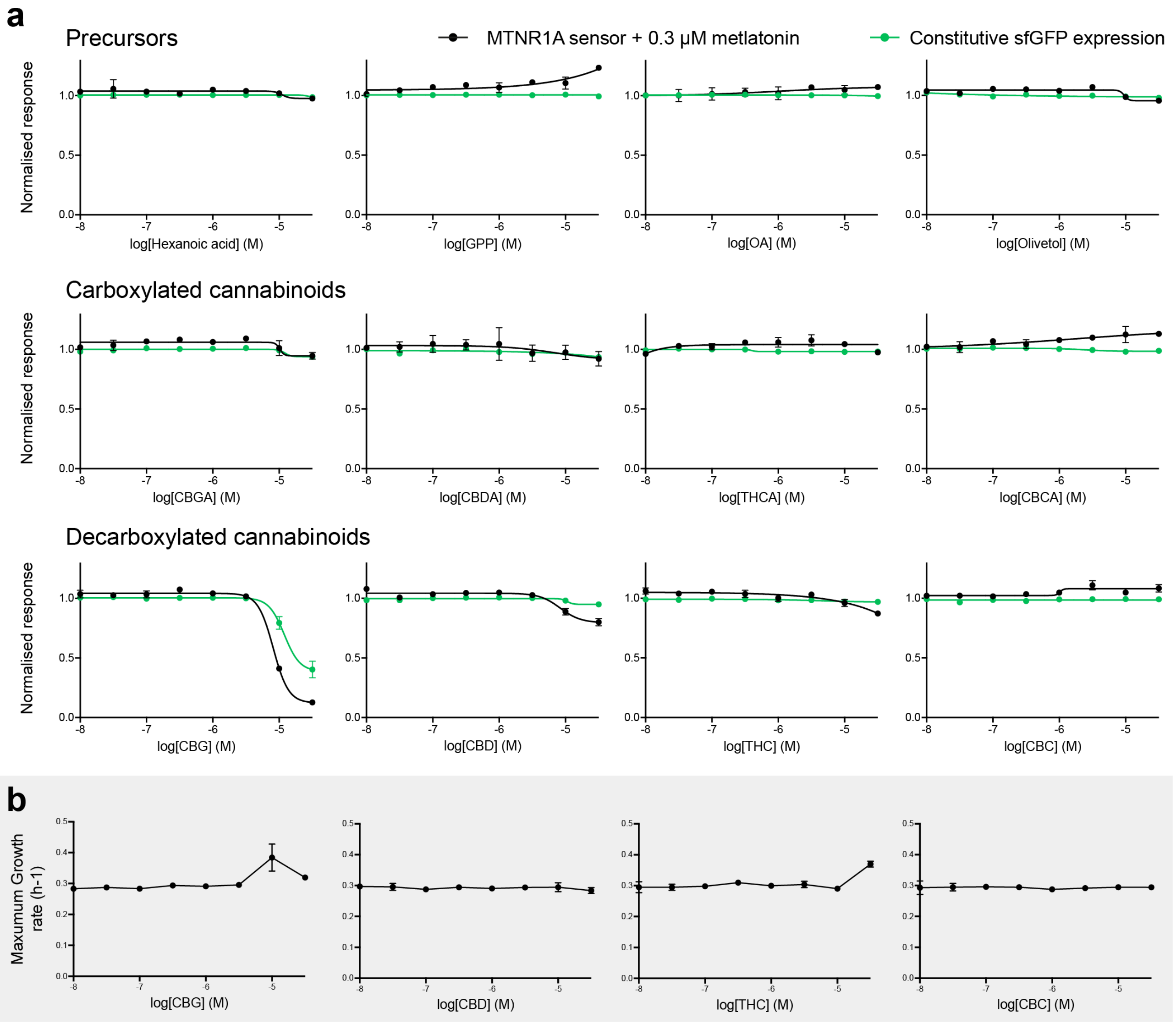

Supplementary Fig. 4. Receptor-independent effects of cannabinoids and precursors. a, Dose-response of the MTNR1A melatonin biosensor (yWS1544, from Shaw et al.) and constitutive GFP expression (BY4741 *pTDH3*-sfGFP-*tTDH1*-*URA3*, this study) to the various cannabinoids and precursors. Black circles and curves are the MTNR1A response at the half maximal-effective concentration (EC_{50}) of melatonin (0.3 μM). Green circles and curve are constitutive GFP expression. Experimental measurements are GFP levels per cell as determined by flow cytometry and shown as the mean ± SD from three biological replicates. All data are normalised to CB2 biosensor response without cannabinoid (1) and untransformed cells (0), and curves were fitted using GraphPad Prism variable slope (four parameter) nonlinear regression fit. **b**, Maximum growth rate of wildtype BY4741 yeast over a range of cannabinoid concentrations. Measurements are maximum growth rate calculated from growth at exponential phase in YPD and shown as mean ± SD from two biological replicates.

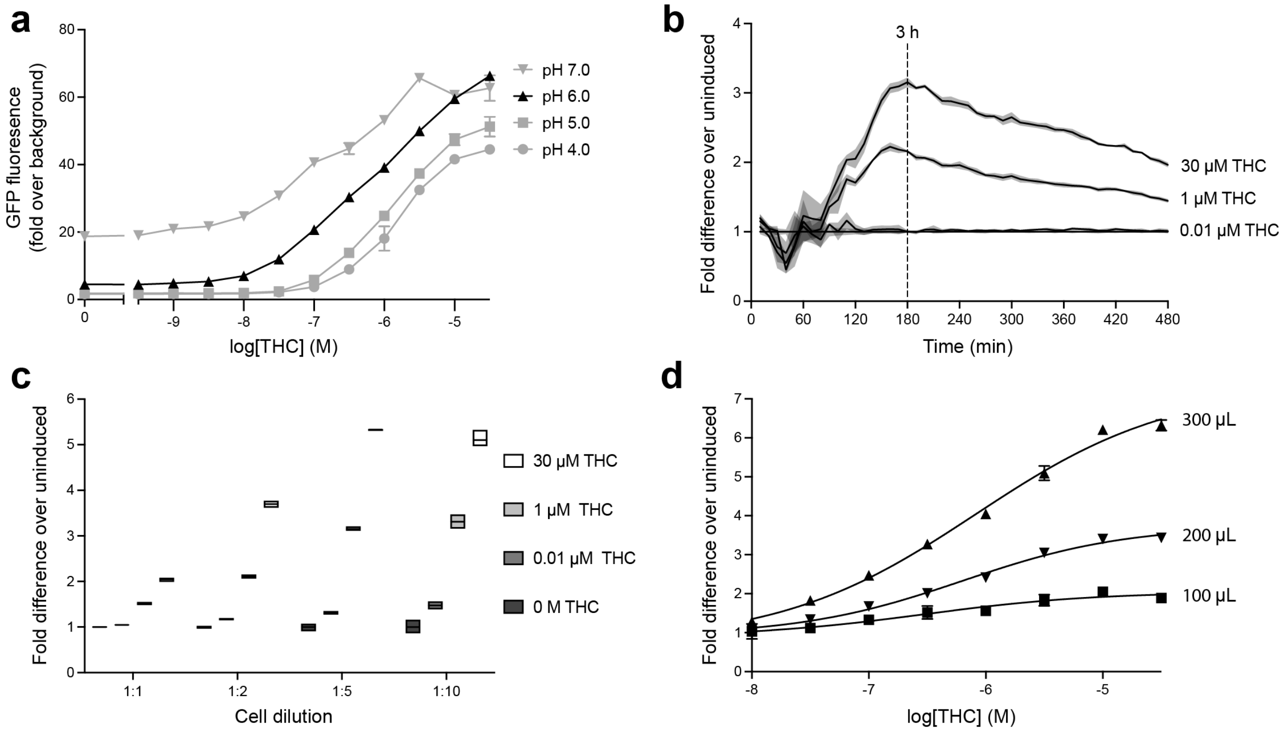

Supplementary Fig. 5. CB2 biosensor plate reader assay optimisation. a, THC dose-response curves with the CB2 biosensor at pH 4 (circles), pH 5 (squares), pH 6 (up triangles), and pH 7 (down triangles). CB2 biosensor behaviour at pH 6 (black) displayed low leak, high sensitivity, and a wide operational range, and so was chosen as the pH for final biosensor assay. Experimental measurements are GFP level per cell as determined by flow cytometry and shown as the mean ± SD from three biological replicates. **b**, Time course of GFP signal after induction with THC. Maximum signal over background (no induction) was seen after 3 h, and so was chosen for the final biosensor assay. **c**, THC dose-response of the CB2 biosensor at different cell dilutions. Diluting cells by resuspending a saturated culture in 5 x fresh media produced the highest signal with lowest error, and so was chosen for the final biosensor assay. **d**, THC dose-response curves of the CB2 biosensor, transferring 100, 200, or 300 μL for measurement in the plate reader. Experimental measurements are GFP level per cell as determined on a plate reader and shown as the mean ± SD from three biological replicates.

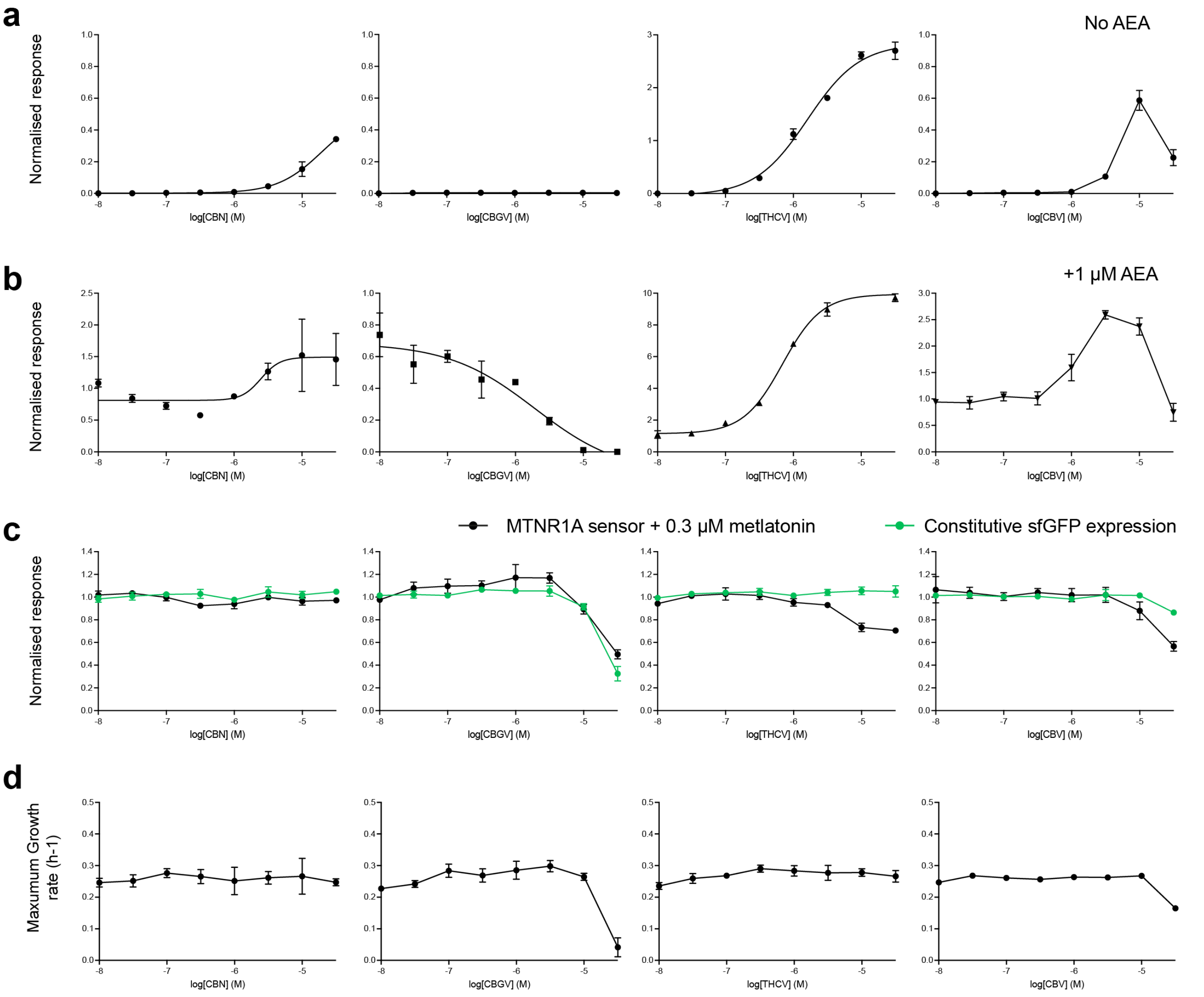

Supplementary Fig. 6. Properties of the oxidised and variant cannabinoids, cannabinol (CBN), cannabigerovarin (CBGV), Δ9-tetrahydrocannabivarin (THCV), and cannabivarin (CBV). **a**, Dose-response curves of cannabinoids with the CB2 biosensor in the absence of AEA. Data normalised to the 30 µM THC response (1). **b**, Dose-response curves of cannabinoids with the CB2 biosensor in the presence of 1 µM AEA. Data normalised to the 1 µM AEA response (1). **c**, Dose-response curves of cannabinoids in the absence of AEA with the MTNR1A biosensor in the presence of 0.3 µM melatonin (black) and constitutive GFP expression (green). Data normalised to the no cannabinoid response (1). Experimental measurements are GFP level per cell determined by flow cytometry and shown as the mean ± SD from three biological replicates. Curves were fitted using GraphPad Prism variable slope (four parameter) nonlinear regression fit for CBN, CBGV, and THCV. **d**, Maximum growth rate of wildtype BY4741 yeast over a range of variant cannabinoid concentrations. Measurements are maximum growth rate calculated from growth at exponential phase in YPD and shown as mean ± SD from three biological replicates.

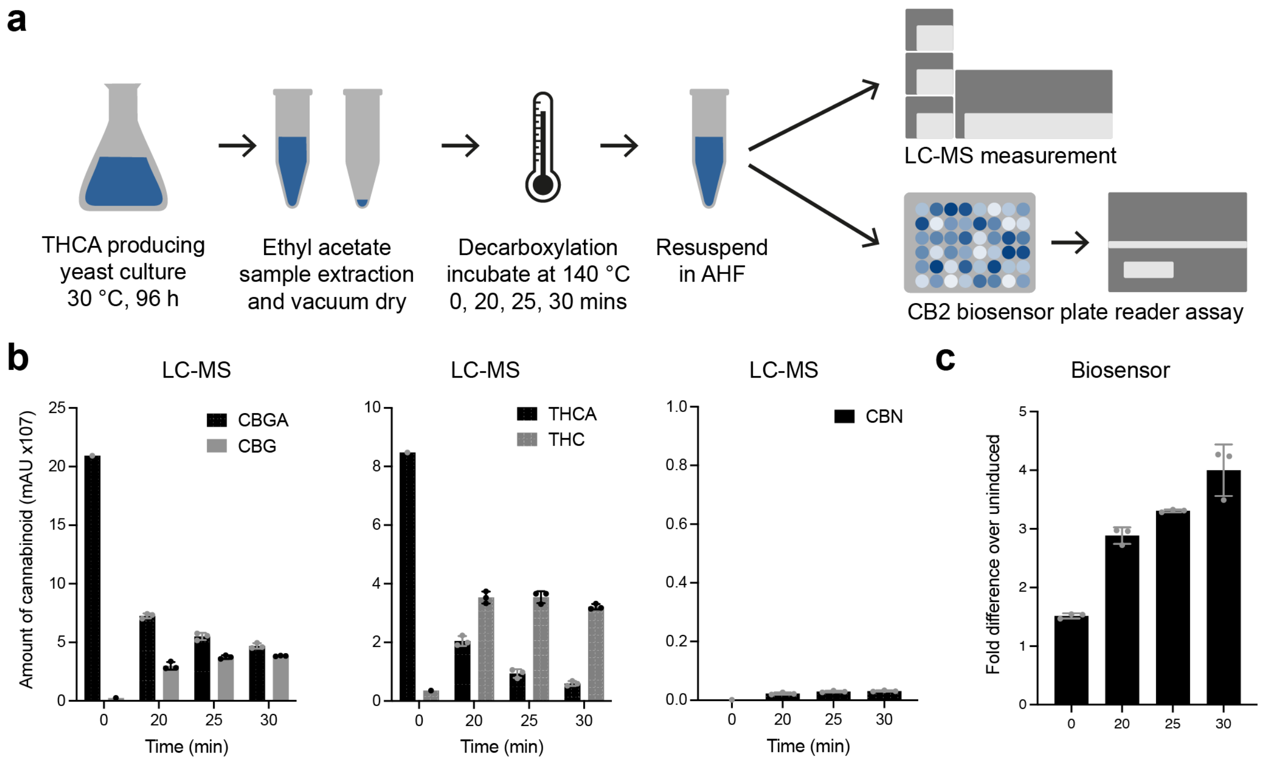

Supplementary Fig. 7. Decarboxylation of THCA producing yeast cell extract. a, Workflow for extracting and decarboxylating acid cannabinoids from yeast and converting them into their non-acid forms and assessment of incubation time at 140 °C by LC-MS and the CB2 biosensor assay. AHF, acetonitrile/H2O/formic acid (80/20/0.05%). **b**, Relative amounts of all cannabinoids detected in cell extract after incubation at 140 °C over time. Experimental measurements are relative amounts as determined by LC-MS and shown as mean ± SD from three technical replicates. **c**, CB2 biosensor response to yeast THCA producer extract after heating at 140 °C for different lengths of time. Data was normalised uninduced CB2 biosensor response (1). Experimental measurements are GFP level per cell as determined on a plate reader and shown as the mean ± SD from three biological replicates.

Note to readers: Different microbial systems, biosynthetic pathways and culture conditions may lead to different ratios of cannabinoids. In this case, the variant cannabinoids are not present, due to the lack of native olivetolic acid biosynthesis. It is recommended that users should recharacterize the optimal time of incubation at 140 °C before undertaking new screening efforts, as this time may between experimental systems. This optimisation can be done using the CB2 biosensor and selecting the time that leads to peak biosensor induction from a control strain that is known to produce THCA.

Supplementary Table 1. THCAS mutant strains in the yS231 library. Colour scale of LC-MS data set to lowest (white) and highest (blue) relative amounts of THC, as a visual aid.

Mutant strain	Codon at AA 415	Amino acid change	Note	LC-MS THC amount (mAU x10^7)	Biosensor read 1 (arb. units)	Biosensor read 2 (arb. units)	Biosensor average (arb. units)
yS231-1	GTA>TAT	V415Y		2.59	1089	1113	1101
yS231-3	GTA>ATG	V415M		4.44	1058	1079	1069
yS231-4	GTA>TTT	V415F		3.13	1134	1130	1132
yS231-5	GTA>AAT	V415N		3.70	1132	1086	1109
yS231-6	GTA>TAT	V415Y		2.24	948	923	935
yS231-7	GTA>TAG	V415*	*, stop codon	1.89	889	871	880
yS231-8	GTA>AAG	V415K		2.52	1014	1014	1014
yS231-9	GTA>AGT	V415S		2.42	961	961	961
yS231-10	GTA>CTG	V415L		2.63	940	942	941
yS231-11	GTA>CTG	V415L		3.50	1174	1136	1155
yS231-12	GTA>TAT	V415Y		1.73	856	823	840
yS231-13	GTA>ACT	V415T		2.73	1009	980	994
yS231-14	GTA>CGT	V415R		1.91	819	811	815
yS231-15	GTA>CAT	V415H		1.84	808	799	804
yS231-16	GTA>TAT	V415Y		1.67	959	931	945
yS231-17	GTA>TGT	V415C		2.28	925	888	906
yS231-18	GTA>CTG	V415L		3.66	1240	1237	1239
yS231-19	GTA>TGT	V415C		3.66	1399	1436	1418
yS231-20	GTA>GTA	V415V	Wild type	3.98	1425	1340	1383
yS231-21	GTA>TCG	V415S		1.94	1060	1083	1072
yS231-22	GTA>CAT	V415H		1.62	862	888	875
yS231-23	GTA>CCT	V415P		1.33	781	812	797
yS231-24	GTA>CCT	V415P		2.18	1048	1085	1066
yS231-25	GTA>ACT	V415T		2.97	1639	1592	1616
yS231-26	GTA>ATT	V415I		3.03	1676	1573	1625
yS231-28	GTA>CCT	V415P		1.68	1327	1322	1325
yS231-29	GTA>CGT	V415R		1.78	1493	1423	1458
yS231-30	GTA>GAG	V415E		2.06	1440	1488	1464
yS231-31	GTA>TAG	V415*	*, stop codon	2.11	1398	1390	1394
yS231-33	GTA>GCT	V415A		2.91	1629	1725	1677
yS231-34	GTA>AAT	V415N		1.67	1193	1154	1174
yS231-35	GTA>AAT	V415N		1.75	1195	1173	1184
yS231-36	GTA>ACG	V415T		2.46	1537	1511	1524
yS231-37	GTA>ACT	V415T		3.39	1773	1697	1735
yS231-39	GTA>CGT	V415R		2.00	1234	1179	1207
yS231-40	GTA>CAT	V415H		1.97	1420	1313	1367
yS231-42	GTA>CCT	V415P		2.10	1208	1181	1194
yS231-43	GTA>CAG	V415Q		2.11	1383	1378	1381
yS231-44	GTA>ACT	V415T		2.00	1462	1445	1454
yS231-45	GTA>TTT	V415F		1.91	1829	1763	1796
yS231-46	GTA>ACG[a]	V415T	[a], AAG>TAG at K457*	2.06	1433	1366	1399
yS231-48	GTA>GTG	V415V	Synonymous codon	3.78	1824	1745	1784
yS231-49	GTA>ACT	V415T		3.09	1497	1540	1518
yS231-51	GTA>TAT	V415Y		1.95	1300	1366	1333
yS231-52	GTA>CAT	V415H		2.29	1592	1620	1606
yS231-53	GTA>AAT	V415N		2.16	1177	1213	1195
yS231-54	GTA>CCG	V415P		1.46	844	852	848
yS231-55	GTA>AAT	V415N		1.03	839	835	837
yS231-56	GTA>TAT[b]	V415Y	[b], AAC>GAC at N514D	1.47	820	827	824
yS231-57	GTA>AAT	V415N		1.42	848	882	865
yS231-58	GTA>GTA	V415V	Wild type	3.21	1169	1168	1169
yS231-59	GTA>CTG	V415L		2.07	900	883	892
yS231-61	GTA>GTT	V415V	Synonymous codon	1.58	851	839	845

yS231-62	GTA>CAG	V415Q		0.98	816	805	810
yS231-63	GTA>CCT	V415P		1.56	939	914	926
yS231-64	GTA>AAG	V415K		2.27	920	875	897
yS231-65	GTA>ACT	V415T		3.00	1085	1066	1075
yS231-68	GTA>TGG	V415W		1.28	820	796	808
yS231-69	GTA>TTG	V415L		1.96	891	909	900
yS231-70	GTA>AAG	V415K		1.60	864	835	849
yS231-71	GTA>GCG	V415A		2.22	929	905	917
yS231-72	GTA>CTG	V415L		1.86	900	894	897
yS231-73	GTA>ATG	V415M		1.46	1030	1008	1019
yS231-74	GTA>GCT	V415A		1.96	1089	1100	1094
yS231-75	GTA>AGG	V415R		1.35	825	843	834
yS231-76	GTA>AAT	V415N		1.57	838	832	835
yS231-77	GTA>TAT	V415Y		1.71	887	909	898
yS231-78	GTA>ACT	V415T		2.06	907	948	928
yS231-79	GTA>CAG	V415Q		1.46	858	887	873
yS231-80	GTA>CCT	V415P		1.38	839	853	846
yS231-81	GTA>GAT	V415D		1.80	868	873	870
yS231-82	GTA>TAT	V415Y		1.46	852	861	856
yS231-83	GTA>TTG	V415L		2.08	939	974	957
yS231-84	GTA>GCT	V415A		2.67	1090	1054	1072
yS231-85	GTA>GCT	V415A		2.63	1099	1114	1106
yS231-87	GTA>ACT	V415T		2.58	1032	1028	1030
yS231-88	GTA>TAG	V415*	*, stop codon	1.69	1035	1015	1025
yS231-89	GTA>TAG	V415*	*, stop codon	1.31	813	810	812
yS231-90	GTA>CAT	V415H		1.53	847	838	843
yS231-91	GTA>TCT	V415S		1.81	850	859	855
yS231-92	GTA>ACT	V415T		1.41	807	821	814
yS231-93	GTA>GTA	V415V	Wild type	2.95	1115	1221	1168
yS231-94	GTA>ATT	V415I		1.56	824	805	814
yS231-95	GTA>CCG	V415P		1.21	812	822	817
yS231-96	GTA>AGT	V415S		1.25	840	823	832
yS231-97	GTA>ATG	V415M		4.89	1568	1595	1582
yS231-98	GTA>TGT	V415C		3.14	892	936	914
yS231-99	GTA>TGT	V415C		4.52	1252	1298	1275
yS231-100	GTA>CAG	V415Q		1.83	867	872	870
yS231-101	GTA>GCG	V415A		2.30	931	987	959
yS231-102	GTA>GTT	V415V	Synonymous codon	2.48	885	929	907
yS231-103	GTA>CTG	V415L		3.40	949	1007	978
yS231-104	GTA>TGG	V415W		4.59	1721	1749	1735
yS231-105	GTA>GAG	V415E		3.90	1015	1049	1032
yS231-106	GTA>TCG	V415S		1.21	858	850	854
yS231-107	GTA>ACG	V415T		2.67	1033	1019	1026
yS231-108	GTA>CAG	V415Q		1.45	848	845	846
yS231-109	GTA>ACG	V415T		2.52	984	947	966
yS231-110	GTA>GTA	V415V	Wild type	5.25	1792	1756	1774
yS231-111	GTA>TAG	V415*	*, stop codon	2.37	950	939	945
yS231-112	GTA>GTA	V415V	Wild type	2.96	1032	1011	1022
yS231-113	GTA>TTG	V415L		1.53	886	884	885
yS231-114	GTA>CAT	V415H		1.27	849	849	849
yS231-115	GTA>AAG	V415K		2.00	957	941	949
yS231-116	GTA>TCG	V415S		1.22	814	839	827
yS231-118	GTA>ATG	V415M		1.91	872	880	876
yS231-119	GTA>AAG	V415K		1.66	855	881	868
yS231-120	GTA>TTG	V415L		2.03	879	910	895

Supplementary Table 2. Ligands used in this study.

Ligand	Abbreviation	Solvent	Supplier	Product code
2-Arachidonoylglycerol	2-AG	Acetonitrile	Sigma	A8973
Anandamide	AEA	Ethanol	Fluorochem	M05752
N-Arachidonylglycine	NaGly	Ethanol	ABCAM	ab120346
L-α-Lysophosphatidylinositol	LPI	DMSO	Sigma	L7635
Oleoylethanolamine	OEA	Ethanol	Sigma	O0383
Hexanoic acid	Hexanoic acid	Methanol	Sigma	153745
Geranyl pyrophosphate	GPP	Methanol	Sigma	G6772-1VL
Divarinic acid	DA	Methanol	TRC	D494463
Olivetolic acid	OA	Methanol	TRC	O533005
Olivetol	Olivetol	Methanol	Sigma	152633
Cannabigerolic acid	CBGA	Acetonitrile	Sigma	C-142-1ML
Cannabidiolic acid	CBDA	Acetonitrile	Sigma	C-144-1ML
Δ^9-Tetrahydrocannabinolic acid	THCA	Acetonitrile	Sigma	T-093-1ML
Cannabichromenic acid	CBCA	Acetonitrile	Sigma	C-150-1ML
Cannabigerol	CBG	Methanol	Sigma	C-141-1ML
Cannabidiol	CBD	Methanol	Sigma	C-045-1ML
Δ^9-Tetrahydrocannabinol	THC	Methanol	Sigma	T4764-1ML
Cannabichromene	CBC	Methanol	Sigma	C-143-1ML
Cannabinol	CBN	Methanol	Sigma	C-046-1ML
Cannabigerovarin	CBGV	Methanol	Sigma	C-227-1ML
Tetrahydrocannabivarin	THCV	Methanol	Sigma	T-094-1ML
Cannabivarin	CBV	Methanol	Sigma	C-225-1ML

Supplementary Table 3. DNA sequences used in this study. All DNA sequences are in the Yeast MoClo Toolkit (YTK) format as a type 3-4a part using the pYTK001 backbone (CamR-ColE1, not shown). BsaI recognition site (grey highlight), BsaI overhang (bold), open reading frame (underlined), start codon (green highlight), stop codon (red highlight).

Name	Insert sequence
**pWS2692**<br><br>**CB1R**	GGTCTCA**TATG**AAGTCTATTTTGGACGGTTTGGCTGATACCACCTTCAGAACTATTACTACCGATTTG<br>TTGTACGTCGGTTCCAACGATATTCAGTACGAAGATATTAAGGGTGACATGGCTTCTAAGTTGGGTT<br>ACTTTCCTCAAAAGTTCCCATTGACATCTTTCAGAGGTTCTCCATTCCAAGAAAAAATGACTGCTGGT<br>GATAACCCACAATTGGTTCCAGCTGATCAAGTTAACATTACCGAGTTCTACAACAAGTCCCTGTCCTC<br>ATTCAAAGAAAACGAAGAAAACATTCAGTGCGGCGAAAACTTCATGGATATTGAATGTTTCATGGTCT<br>TGAACCCATCTCAACAATTGGCTATTGCTGTTTTGTCTTTGACCTTGGGTACTTTCACTGTCTTGGAA<br>AACTTGTTGGTTTTGTGCGTTATCTTGCACTCCAGATCTTTGAGATGTAGACCATCCTACCATTTCATT<br>GGTTCTTTGGCTGTTGCAGATTTGTTGGGTTCTGTTATTTTCGTCTACTCCTTCATCGATTTCCACGTT<br>TTCCATAGAAAGGACTCCAGAAACGTTTTCTTGTTCAAGTTAGGTGGTGTTACTGCTTCTTTCACTGC<br>TTCTGTTGGTTCCTTGTTTTTGACTGCTATTGACAGGTACATCTCCATCCATAGACCATTGGCTTACA<br>AGAGAATAGTTACTAGACCAAAGGCTGTTGTTGCTTTCTGTTTGATGTGGACTATTGCTATCGTTATT<br>GCAGTCTTGCCATTGCTAGGTTGGAATTGTGAAAAGTTGCAATCCGTTTGCTCCGATATTTTCCCACA<br>TATTGACGAAACCTACCTGATGTTTTGGATTGGTGTTACCTCTGTCTTGTTGTTGTTTATCGTTTACGC<br>CTACATGTACATTTTGTGGAAGGCTCATTCTCATGCCGTTAGAATGATTCAAAGAGGTACTCAGAAGT<br>CGATCATCATCCATACTTCAGAAGATGGTAAGGTTCAAGTCACTAGACCAGATCAAGCTAGAATGGA<br>TATTAGATTGGCTAAGACCTTGGTCTTGATCTTGGTTGTTTTGATTATTTGCTGGGGTCCTTTGTTGG<br>CCATTATGGTTTATGATGTTTTCGGCAAGATGAACAAGCTGATTAAGACTGTTTTCGCCTTCTGTTCT<br>ATGCTGTGTTTGTTGAACTCTACTGTCAACCCAATTATCTACGCCTTGAGATCTAAGGATTTGAGACA<br>TGCCTTTAGGTCTATGTTCCCATCTTGTGAAGGTACTGCTCAACCATTGGATAATTCTATGGGTGATT<br>CTGACTGCTTGCACAAACATGCTAACAATGCTGCTTCAGTTCATAGAGCTGCTGAATCTTGTATTAAG<br>TCCACCGTTAAGATTGCCAAGGTTACCATGTCTGTTTCTACTGATACTTCTGCTGAAGCCTTGTAACT<br>CGAG**TGGC**TGAGACC
**pWS2693**<br><br>**CB2R**	GGTCTCA**TATG**GAAGAGTGTTGGGTTACTGAAATTGCCAACGGTTCTAAAGACGGTTTGGATTCTAA<br>TCCCATGAAGGACTACATGATTTTGTCTGGTCCACAAAAAACTGCTGTTGCTGTTTTGTGTACTTTGT<br>TGGGTTTGTTGTCTGCCTTGGAAAACGTTGCTGTCCTGTACTTGATTCTGTCCTCTCATCAATTGAGA<br>AGAAAGCCCTCTTACTTGTTCATTGGTTCTTTGGCTGGTGCTGATTTTTTGGCTTCTGTTGTTTTTGCT<br>TGCTCCTTCGTTAACTTCCATGTTTTCCATGGTGTTGATTCCAAGGCTGTTTTCTTGTTGAAGATTGG<br>TTCTGTCACTATGACTTTCACTGCTTCTGTAGGTTCTTTGTTGTTGACCGCTATTGATAGATACCTGT<br>GTTTGAGATATCCACCATCTTACAAGGCTTTGTTGACTAGAGGTAGAGCTTTGGTTACTTTGGGTATT<br>ATGTGGGTTTTGTCCGCTTTGGTTTCTTACTTGCCATTGATGGGTTGGACTTGTTGTCCAAGACCATG<br>TTCTGAATTATTCCCATTGATCCCAAACGACTACTTGTTGAGTTGGTTGTTGTTTATCGCCTTCTTGTT<br>CTCCGGTATTATCTACACTTATGGTCACGTTTTGTGGAAGGCTCATCAACACGTTGCTTCATTATCTG<br>GTCATCAAGATAGACAAGTTCCAGGTATGGCTAGAATGAGATTGGATGTTAGATTGGCTAAGACCTT<br>GGGTTTAGTTTTGGCCGTTTTGTTGATTTGTTGGTTCCCAGTTTTGGCTTTGATGGCTCATTCTTTAG<br>CTACTACCTTGTCCGATCAAGTTAAGAAGGCTTTTGCTTTCTGTTCCATGTTGTGCTTGATCAACTCT<br>ATGGTTAACCCAGTTATCTACGCTTTGAGATCCGGTGAAATTAGATCTTCTGCTCATCATTGCTTGGC<br>CCATTGGAAAAAATGTGTTAGAGGTTTGGGTTCCGAAGCAAAAGAAGAAGCTCCAAGATCTTCAGTT<br>ACAGAAACTGAAGCTGATGGTAAGATTACTCCATGGCCAGATTCTAGAGATTTGGATTTGTCTGATTG<br>TTAACTCGAG**TGGC**TGAGACC
**pWS2694**<br><br>**GPR55**	GGTCTCA**TATG**TCTCAACAAAACACTTCTGGTGATTGCTTGTTCGACGGTGTTAACGAATTGATGAAA<br>ACCTTGCAATTCGCCGTTCATATTCCAACTTTTGTCTTGGGTTTGCTGTTGAACTTGTTGGCTATTCA<br>CGGTTTCTCCACGTTCTTGAAGAATAGATGGCCAGATTATGCTGCTACCTCTATCTACATGATTAACT<br>TGGCTGTTTTCGACCTGTTGTTGGTTTTGTCTTTGCCATTCAAAATGGTCTTGTCCCAAGTCCAATCT<br>CCATTTCCATCTTTGTGTACTTTGGTCGAATGCCTGTACTTCGTTTCTATGTACGGTTCTGTTTTCACC<br>ATCTGCTTCATCTCTATGGATAGATTCTTGGCCATTAGATACCCACTGTTGGTTTCTCATTTGAGATC<br>CCCAAGAAAGATTTTCGGTATTTGTTGCACCATCTGGGTTTTAGTTTGGACTGGTTCTATTCCCATCT<br>ACTCATTTCATGGTAAGGTCGAGAAGTACATGTGCTTCCATAATATGTCTGATGATACCTGGTCTGCC<br>AAGGTGTTTTTTCCATTGGAAGTTTTCGGTTTCTTGCTGCCAATGGGTATTATGGGTTTTTGTTGCTC<br>CAGATCCATCCATATTTTGTTGGGTAGAAGAGATCATACCCAAGATTGGGTTCAACAAAAGGCTTGC<br>ATATACTCTATTGCTGCTTCTTTGGCTGTGTTCGTTGTTTCATTTTTGCCAGTTCACTTGGGCTTTTTC<br>TTGCAATTTTTGGTGAGGAACTCCTTCATCGTTGAATGTAGAGCTAAGCAGTCTATCTCCTTCTTCTT<br>GCAACTGTCTATGTGCTTCTCTAACGTTAACTGTTGCTTGGATGTTTTCTGCTACTACTTCGTCATCA<br>AAGAATTCAGGATGAACATCAGAGCCCATAGACCATCTAGAGTTCAATTGGTTTTACAAGACACCAC<br>CATCTCTAGAGGTTAACTCGAG**TGGC**TGAGACC

**pWS2695**<br><br>**GPR18**	GGTCTCA**TATG**ATTACCTTGAACAATCAAGATCAGCCCGTTCCATTCAATTCTTCTCATCCAGACGAG<br>TACAAGATTGCTGCTTTGGTTTTCTACTCCTGCATTTTCATTATCGGCTTGTTCGTTAACATTACCGCT<br>TTGTGGGTTTTCTCTTGTACCACCAAAAAGAGAACTACCGTTACCATCTACATGATGAATGTTGCCTT<br>GGTTGACCTGATTTTCATCATGACTTTGCCATTCAGGATGTTCTACTACGCTAAAGATGAATGGCCTT<br>TCGGTGAATACTTCTGCCAAATTTTGGGTGCTTTGACTGTTTTCTACCCATCTATTGCTTTGTGGTTG<br>TTGGCTTTCATTTCTGCTGATAGATATATGGCCATCGTTCAACCTAAATACGCCAAAGAGTTGAAGAA<br>CACTTGTAAGGCTGTTTTGGCTTGTGTTGGTGTTTGGATTATGACTTTGACTACTACTACCCCTCTGC<br>TGTTGTTGTACAAAGATCCAGATAAGGATTCTACTCCAGCTACCTGTTTGAAGATTTCCGATATTATC<br>TACTTGAAGGCCGTTAACGTTTTGAACTTGACTAGATTGACCTTCTTCTTCTTGATCCCCTTGTTCATT<br>ATGATCGGTTGCTACTTGGTTATCATCCACAATTTGTTGCATGGTAGGACCTCTAAATTGAAGCCAAA<br>GGTCAAAGAAAAGTCCATCAGAATCATCATCACCTTGTTGGTTCAAGTTTTGGTTTGCTTCATGCCAT<br>TCCATATTTGCTTCGCTTTCTTGATGTTAGGTACTGGTGAAAACTCTTACAATCCATGGGGTGCTTTT<br>ACTACCTTCTTGATGAATTTGTCTACCTGCTTGGATGTCATCCTGTACTACATCGTTTCTAAGCAATTC<br>CAAGCCAGAGTTATCTCCGTTATGTTGTACAGAAACTACCTGAGGTCTATGCGTAGAAAGTCTTTTAG<br>ATCAGGTTCCTTGAGGTCCTTGTCCAACATTAACTCTGAAATGCTGTAACTCGAG**TGGC**TGAGACC
**pWS2696**<br><br>**GPR119**	GGTCTCA**TATG**GAATCTTCATTTTCTTTCGGTGTTATCTTGGCTGTTTTGGCCTCTTTGATTATTGCTA<br>CCAATACCTTGGTTGCTGTTGCCGTTTTGTTGTTGATTCATAAGAACGACGGTGTCTCTTTGTGCTTC<br>ACTTTGAATTTGGCTGTTGCTGATACCTTGATCGGTGTTGCTATTTCTGGTTTGTTGACCGATCAATT<br>GTCATCTCCATCAAGACCAACTCAAAAGACCTTGTGTTCTTTGAGAATGGCTTTCGTTACTTCTTCAG<br>CTGCTGCTTCTGTTTTGACCGTTATGTTGATTACCTTCGATAGATACTTGGCCATCAAGCAACCATTC<br>AGATACTTGAAGATTATGTCCGGTTTTGTTGCTGGTGCTTGTATTGCTGGTTTATGGTTGGTTTCTTA<br>CTTGATTGGCTTTTTGCCATTGGGTATTCCAATGTTCCAACAAACTGCTTACAAGGGTCAATGTTCTT<br>TCTTCGCTGTTTTCCATCCACATTTCGTTTTGACCTTGTCTTGCGTTGGTTTTTTTCCAGCTATGCTGT<br>TGTTCGTTTTCTTCTACTGTGACATGTTGAAGATCGCCTCTATGCATTCCCAACAGATTAGAAAAATG<br>GAACATGCTGGTGCAATGGCTGGTGGTTATAGATCACCAAGAACTCCATCTGATTTCAAGGCTTTGA<br>GAACTGTTTCAGTGTTGATTGGTTCTTTCGCTTTGTCTTGGACTCCATTCTTGATTACTGGTATCGTTC<br>AAGTTGCTTGTCAAGAATGTCACTTGTACTTGGTCTTGGAAAGATACTTGTGGTTGTTAGGTGTTGGC<br>AACTCTTTGTTGAATCCATTGATCTATGCCTACTGGCAGAAAGAAGTCAGATTGCAGTTGTATCATAT<br>GGCCTTGGGTGTTAAGAAGGTCTTGACATCTTTCCTGTTGTTCTTGTCTGCTAGAAATTGTGGTCCA<br>GAAAGACCAAGAGAATCTTCTTGTCATATCGTCACCATCTCCAGCTCTGAATTTGATGGTTAACTCGA<br>G**TGGC**TGAGACC

Supplementary Note 1. Gating strategy for flow cytometry.

Yeast cells were gated for singlets using FSC-H vs FSC-A and to remove background noise. No other Gating was performed on global yeast population. > 10,000 events were collected and analysed within the singlets gate for each measurement.

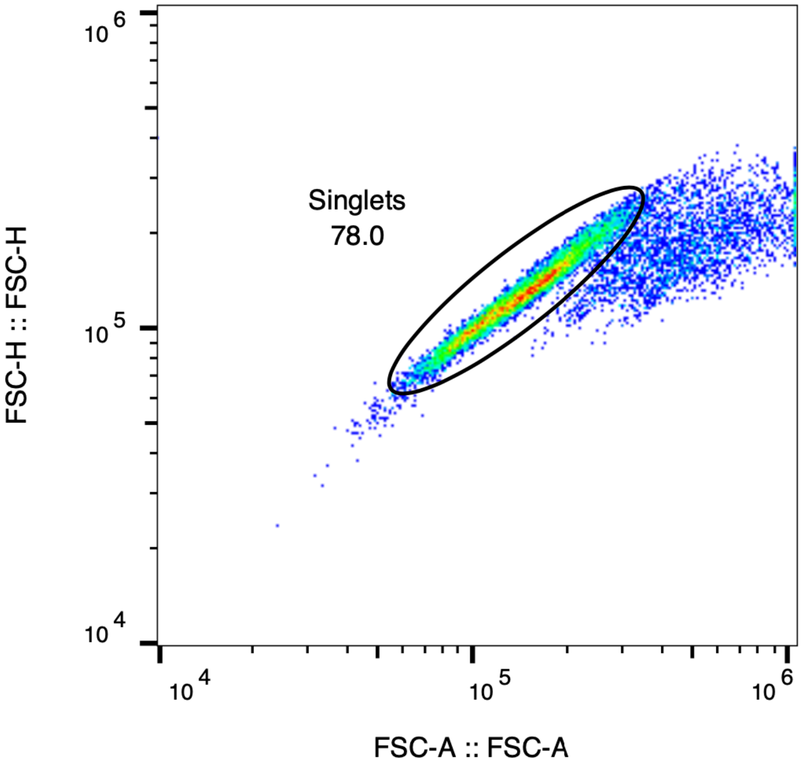

Supplement: Supplementary file 1 — Supplementary Information [file 41467_2022_33207_MOESM1_ESM.pdf]
