# Supplementary material for: Screening microbially produced Δ9-tetrahydrocannabinol using a yeast biosensor workflow
Source: Nat Commun. 2022 Sep 20;13:5509. doi: 10.1038/s41467-022-33207-x (PMC9489785; doi:10.1038/s41467-022-33207-x)
Supplement: Supplementary file 3 — Reporting Summary [file 41467_2022_33207_MOESM3_ESM.pdf]

nature research

Corresponding author(s): Tom Ellis

Last updated by author(s): 16/8/2022



# Reporting Summary

Nature Research wishes to improve the reproducibility of the work that we publish. This form provides structure for consistency and transparency in reporting. For further information on Nature Research policies, see our Editorial Policies and the Editorial Policy Checklist.

## Statistics

For all statistical analyses, confirm that the following items are present in the figure legend, table legend, main text, or Methods section.

| n/a | Confirmed | |
|---|---|---|
| ☐ | ☒ | The exact sample size ($n$) for each experimental group/condition, given as a discrete number and unit of measurement |
| ☐ | ☒ | A statement on whether measurements were taken from distinct samples or whether the same sample was measured repeatedly |
| ☐ | ☒ | The statistical test(s) used AND whether they are one- or two-sided<br>*Only common tests should be described solely by name; describe more complex techniques in the Methods section.* |
| ☒ | ☐ | A description of all covariates tested |
| ☒ | ☐ | A description of any assumptions or corrections, such as tests of normality and adjustment for multiple comparisons |
| ☐ | ☒ | A full description of the statistical parameters including central tendency (e.g. means) or other basic estimates (e.g. regression coefficient) AND variation (e.g. standard deviation) or associated estimates of uncertainty (e.g. confidence intervals) |
| ☐ | ☒ | For null hypothesis testing, the test statistic (e.g. $F$, $t$, $r$) with confidence intervals, effect sizes, degrees of freedom and $P$ value noted<br>*Give P values as exact values whenever suitable.* |
| ☒ | ☐ | For Bayesian analysis, information on the choice of priors and Markov chain Monte Carlo settings |
| ☒ | ☐ | For hierarchical and complex designs, identification of the appropriate level for tests and full reporting of outcomes |
| ☐ | ☒ | Estimates of effect sizes (e.g. Cohen's $d$, Pearson's $r$), indicating how they were calculated |

*Our web collection on statistics for biologists contains articles on many of the points above.*

## Software and code

Policy information about availability of computer code

| | |
|---|---|
| Data collection | 1. Attune NxT software v3.1 was used to collect flow cytometry data.<br>2. Molecular Devices Softmax Pro v7.1 was used to collect plate reader data (SpectraMax plate reader)<br>3. i.control v2.0 was used to collect plate reader data (Tecan Infinite 200 PRO plate reader)<br>4. Agilent MassHunter v10 was used to collect LC-MS data. |
| Data analysis | FlowJo v10.6 was used to analyse flow cytometry data.<br>Agilent MassHunter Qualitative Analysis B.06.00 was used to analyse flow cytometry data.<br>GraphPad Prism 9 was used to generate all graphs, plot curves, and statistical analysis.<br>MicroSoft Excel v16 was used for analysing plate reader data. |

For manuscripts utilizing custom algorithms or software that are central to the research but not yet described in published literature, software must be made available to editors and reviewers. We strongly encourage code deposition in a community repository (e.g. GitHub). See the Nature Research guidelines for submitting code & software for further information.

## Data

Policy information about availability of data

All manuscripts must include a data availability statement. This statement should provide the following information, where applicable:

- Accession codes, unique identifiers, or web links for publicly available datasets
- A list of figures that have associated raw data
- A description of any restrictions on data availability

Nucleotide sequence data of all G protein-coupled receptors used in this study are included in Supplementary Table 2. Nucleotide sequence data for all other GPCR biosensor components previously reported in Shaw et al. (2019)16. Nucleotide sequence data for all cannabinoid producing strains previously reported in Luo et al.

(2019)9. The CB2 biosensor strain will be made available from the corresponding author. Strains producing controlled substances or direct precursors of controlled substances can only be provided to laboratories/institutions with appropriate approvals and licenses. Individual data points for all graphs are provided as source data with this paper.

# Field-specific reporting

Please select the one below that is the best fit for your research. If you are not sure, read the appropriate sections before making your selection.

- [x] Life sciences
- [ ] Behavioural & social sciences
- [ ] Ecological, evolutionary & environmental sciences

For a reference copy of the document with all sections, see nature.com/documents/nr-reporting-summary-flat.pdf

# Life sciences study design

All studies must disclose on these points even when the disclosure is negative.

| | |
|---|---|
| Sample size | No sample size calculation was performed. Based on previous publications (e.g. Shaw et al. 2019, https://doi.org/10.1016/j.cell.2019.02.023), experiments in were performed in triplicates (n = 3) or greater. |
| Data exclusions | No data were excluded from the manuscript. |
| Replication | All experiments were performed in duplicates, triplicates, or quadruplicates and all attempts at replication were successful (size indicated in figure legend). |
| Randomization | Transformed yeast colonies were chosen at random from plates and no data was excluded. |
| Blinding | The study does not contain experiments where blinding would be applicable. |

# Reporting for specific materials, systems and methods

We require information from authors about some types of materials, experimental systems and methods used in many studies. Here, indicate whether each material, system or method listed is relevant to your study. If you are not sure if a list item applies to your research, read the appropriate section before selecting a response.

## Materials & experimental systems

| n/a | Involved in the study | |
|---|---|---|
| ☒ | ☐ | Antibodies |
| ☐ | ☒ | Eukaryotic cell lines |
| ☒ | ☐ | Palaeontology and archaeology |
| ☒ | ☐ | Animals and other organisms |
| ☒ | ☐ | Human research participants |
| ☒ | ☐ | Clinical data |
| ☒ | ☐ | Dual use research of concern |

## Methods

| n/a | Involved in the study | |
|---|---|---|
| ☒ | ☐ | ChIP-seq |
| ☐ | ☒ | Flow cytometry |
| ☒ | ☐ | MRI-based neuroimaging |

# Eukaryotic cell lines

Policy information about cell lines

| | |
|---|---|
| Cell line source(s) | Saccharomyces cerevisiae Strain yWS677 from Tom Ellis lab (published in Shaw et al 2019 DOI: 10.1016/j.cell.2019.02.023) Saccharomyces cerevisiae Strain yCAN31 from Synthetic Biology Engineering Research Center (Synberc) Registry (https://synberc-registry.jbei.org/) |
| Authentication | We confirmed all derivative strains by PCR and sequencing. |
| Mycoplasma contamination | Yeast does not have this contamination. |
| Commonly misidentified lines (See ICLAC register) | No common misidentified lines were used. |

# Flow Cytometry

## Plots

Confirm that:

- [x] The axis labels state the marker and fluorochrome used (e.g. CD4-FITC).
- [x] The axis scales are clearly visible. Include numbers along axes only for bottom left plot of group (a 'group' is an analysis of identical markers).
- [x] All plots are contour plots with outliers or pseudocolor plots.
- [x] A numerical value for number of cells or percentage (with statistics) is provided.

## Methodology

| | |
|---|---|
| Sample preparation | Cells were measured at approx. OD600 = 0.6 directly from SC medium with no dilution. |
| Instrument | Attune NxT 3 colour with Autosampler |
| Software | Attune NxT software for collection. FlowJo Version 10.6 for data analysis. |
| Cell population abundance | Typical samples included at least 10,000 cells. |
| Gating strategy | Cells were grown in mid log and gated for singlets by FSC-H vs FSC-A and to remove cellular debris. No other Gating was performed on global yeast population. |

- [x] Tick this box to confirm that a figure exemplifying the gating strategy is provided in the Supplementary Information.
